# Clinical Benefits of Laparoscopic Adhesiolysis during Peritoneal Dialysis Catheter Insertion: A Single-Center Experience

**DOI:** 10.3390/medicina59061014

**Published:** 2023-05-24

**Authors:** Hao-Wei Kou, Chun-Nan Yeh, Chun-Yi Tsai, Shou-Hsuan Liu, Wen-Yu Ho, Chao-Wei Lee, Shang-Yu Wang, Ming-Yang Chang, Ya-Chung Tian, Jun-Te Hsu, Tsann-Long Hwang

**Affiliations:** 1 Division of General Surgery, Department of Surgery, Chang Gung Memorial Hospital, Linkou Medical Center, Taoyuan 33305, Taiwan; 2 Department of Nephrology, Chang Gung Memorial Hospital, College of Medicine, Chang Gung University, Taoyuan 33305, Taiwan

**Keywords:** catheter insertion, intra-abdominal adhesions, laparoscopic adhesiolysis, mechanical obstruction, overall catheter survival, peritoneal dialysis, peritoneal dialysis adequacy

## Abstract

*Background and Objectives:* In peritoneal dialysis (PD) therapy, intra-abdominal adhesions (IAAs) can cause catheter insertion failure, poor dialysis function, and decreased PD adequacy. Unfortunately, IAAs are not readily visible to currently available imaging methods. The laparoscopic approach for inserting PD catheters enables direct visualization of IAAs and simultaneously performs adhesiolysis. However, a limited number of studies have investigated the benefit/risk profile of laparoscopic adhesiolysis in patients receiving PD catheter placement. This retrospective study aimed to address this issue. *Materials and Methods:* This study enrolled 440 patients who received laparoscopic PD catheter insertion at our hospital between January 2013 and May 2020. Adhesiolysis was performed in all cases with IAA identified via laparoscopy. We retrospectively reviewed data, including clinical characteristics, operative details, and PD-related clinical outcomes. *Results:* These patients were classified into the adhesiolysis group (*n* = 47) and the non-IAA group (*n* = 393). The clinical characteristics and operative details had no remarkable between-group differences, except the percentage of prior abdominal operation history was higher and the median operative time was longer in the adhesiolysis group. PD-related clinical outcomes, including incidence rate of mechanical obstruction, PD adequacy (Kt/V urea and weekly creatinine clearance), and overall catheter survival, were all comparable between the adhesiolysis and non-IAA groups. None of the patients in the adhesiolysis group suffered adhesiolysis-related complications. *Conclusions*: Laparoscopic adhesiolysis in patients with IAA confers clinical benefits in achieving PD-related outcomes comparable to those without IAA. It is a safe and reasonable approach. Our findings provide new evidence to support the benefits of this laparoscopic approach, especially in patients with a risk of IAAs.

## 1. Introduction

Peritoneal dialysis (PD) is a widely used treatment modality for end-stage renal disease (ESRD) [1]. PD relies on the peritoneum as a dialysis membrane to eliminate waste products and excess water from the body [2]. Therefore, an intact peritoneal membrane and sufficient abdominal cavity space are crucial for well-functioning dialysis. Intra-abdominal adhesions (IAAs) are fibrous bands that form between abdominal organs and the peritoneum [3,4]. IAAs are a common occurrence, following not only abdominal surgery but also inflammation, infection, and radiation therapy [3,4,5]. In PD therapy, IAAs can cause catheter malposition, resulting in flow dysfunction, limited peritoneal surface area, and impeded drainage due to compartmentalization, leading to catheter insertion failure, poor dialysis function, and decreased PD adequacy [6,7,8,9,10,11]. Additionally, IAAs may increase the risk of several intraoperative complications when blind PD catheter insertion is performed using traditional open or percutaneous insertion methods [12,13,14]. Unfortunately, IAAs are usually asymptomatic, have no characteristic laboratory features, and are not readily detected by currently available imaging methods [4]. Although prior abdominal surgery is a risk factor for IAAs, not all patients with IAAs have such a history [5,6,14,15]. Thus, it is an imperative issue to detect and treat preexisting IAAs in patients who have chosen PD as their dialysis modality.

Over the last few decades, the laparoscopic approach has become a popular method for inserting PD catheters [8,9,13]. One of the advantages of laparoscopy is that it enables direct visualization of IAAs, assessment of IAA extent, and simultaneously performs adhesiolysis, which is an adjunctive procedure of the advanced laparoscopic technique [8,9,13,16]. Current guidelines recommend that laparoscopic adhesiolysis should be performed during PD catheter implantation to reduce catheter dysfunction [8,13]. However, many studies only reported the number of patients receiving laparoscopic adhesiolysis without exploring its benefits [12,14,17,18,19]. Other studies compared catheter function or survival between two study groups (e.g., basic vs. advanced laparoscopy or prior abdominal surgery vs. no surgery), but the benefit of several adjunctive procedures, such as adhesiolysis, hernia repair, and omentopexy, was evaluated together [20,21,22,23], thus, not demonstrating the sole effect of adhesiolysis. Only one study reported that patients requiring adhesiolysis had a lower survival probability free from catheter obstruction but had a similar long-term catheter survival compared with patients not requiring adhesiolysis [11]. Accordingly, more evidence regarding the clinical benefits of adhesiolysis is needed to support the guidelines’ recommendation.

In this retrospective study, we aimed to assess the benefit/risk profile of laparoscopic adhesiolysis in patients with IAAs identified during laparoscopic PD catheter placement. We compared the incidence rate of mechanical obstruction, overall catheter survival, and PD adequacy between the adhesiolysis and non-IAA groups during a follow-up period.

## 2. Materials and Methods

### 2.1. Patient Population

This retrospective study was approved by the Institutional Review Board of Chang Gung Memorial Hospital (approval number 202300642B0). Per our institutional guidelines, the need for informed consent was waived for this retrospective study. From January 2013 to May 2020, patients who received laparoscopic PD catheter insertion at Linkou Chang Gung Memorial Hospital were identified, and their data were extracted from the institution’s database. Patients who underwent traditional open PD catheter placement, those under 18 years of age, and those who did not complete the first PD adequacy test were excluded. This study included 440 patients, and adhesiolysis was performed in all cases with IAAs.

### 2.2. Definitions and Data Collection

The patients were divided into the adhesiolysis group and non-IAA group for analysis based on the surgical procedure. We retrospectively reviewed each patient’s demographic characteristics, preoperative laboratory examinations, operative details, complications, and outcomes. The diagnosis of catheter obstruction was established if surgical intervention or catheter removal was required. We defined overall catheter survival as the time from PD catheter insertion to PD discontinuation or the date of the last follow-up without an event. We evaluated PD adequacy through two tests performed 12 weeks and 6 months after commencing PD. PD adequacy was assessed via weekly normalized urea clearance (Kt/V urea) and weekly creatinine clearance (WCCr) [24].

### 2.3. Laparoscopic Surgery and Adhesiolysis

The patients were placed in the supine position and received prophylactic antibiotics before surgery. Under general anesthesia, an incision was made below the umbilicus, and pneumoperitoneum was established. An 11 mm subumbilical incision was made, and the pneumoperitoneum was increased to 12 mm Hg. An 11 mm trocar was inserted in the incision, and then a videoscope was inserted. Another 3 mm port was inserted at the left or right pararectus line at the proper height under direct visualization. Then, we switched the scope to the 3 mm trocar. At first, the patient was placed in the Trendelenberg position. The abdominal cavity was fully examined, and laparoscopic adhesiolysis was performed if IAAs were identified in the lower abdomen/pelvic region. The adhesiolysis was achieved using a combination of gentle blunt and sharp dissection with scissors. Electrocoagulation was used for dense adhesions or areas with vascularity. Care was taken to avoid visceral organ injury or extensive damage to the integrity of the peritoneal membrane during the dissection. The severity of IAAs was classified into four categories, as follows: Grade 0 (no adhesions), Grade 1 (flimsy thickness, avascular), Grade 2 (moderate thickness, limited vascularity), and Grade 3 (dense thickness, vascularized) [25]. After adhesiolysis, we performed prophylactic omentopexy by fixing the inferior edge of the omentum to the round ligament of the liver using titanium clips. The fixation was repeated three to four times in different directions to keep the omentum in the upper abdominal cavity. After that, we routinely performed internal fixation of the catheter to prevent catheter migration. One 3-0 Nylon suture with a straight needle was inserted into the inner opening of the catheter after one thread of suture was passed through the side hole of the catheter. Then, the catheter was introduced through the 11 mm trocar, and its internal tip was brought to the medial umbilical ligament. The straight needle was passed through the medial umbilical ligament under direct vision to prevent vessel injury. Then, the catheter was fixed to the medial umbilical ligament after the two threads of suture were fixed using titanium clips. We also examined the presence of occult hernia or patent processus vaginalis through laparoscopy. Subsequent hernia repair was performed in the presence of hernia. These procedures were described in detail in our previous studies [26,27,28]. Following these adjunctive procedures, the external end of the catheter was brought out from the infraumbilical wound, and subsequent deep cuff fixation and subcutaneous tunneling toward the exit site were completed as usual.

### 2.4. Statistical Analysis

All statistical analyses were conducted using IBM SPSS Statistics (version 21). Categorical variables were analyzed using Pearson’s χ^2^ test or Fisher’s exact test and presented as frequencies and percentages. Continuous variables were assessed for normality using the Kolmogorov–Smirnov test. Normally distributed continuous variables were compared using Student’s *t*-test and were expressed as mean ± standard deviation, while non-normally distributed continuous variables were compared using the Mann–Whitney U test and presented as medians with interquartile ranges. Time-to-event data were analyzed using Kaplan–Meier curve analysis followed by the log-rank test to determine differences between the two study groups. A *p*-value less than 0.05 was considered statistically significant.

## 3. Results

### 3.1. Characteristics of Patients

This retrospective study enrolled 440 patients (225 males and 215 females) who underwent laparoscopic PD catheter placement. The mean age of our patient population was 51.0 ± 15.2 (range, 18–93) years. The mean follow-up time was 46.1 ± 27.5 (range, 3.6–118.9) months. Forty-seven (10.7%) patients had IAAs and received laparoscopic adhesiolysis; 26, 17, and 4 patients had IAAs graded 1, 2, and 3, respectively. Among these 47 patients, 29 (61.7%) had a history of previous abdominal surgery; the types of prior surgery are listed in Table 1. The patients were then categorized into the adhesiolysis group (n = 47) and the non-IAA group (n = 393).

### 3.2. Comparisons of Clinical Characteristics and Preoperative Laboratory Data

Table 2 shows comparisons of clinical characteristics and preoperative laboratory data between two study groups. As shown, the patients in the adhesiolysis group were older (57.7 ± 14.0 vs. 50.2 ± 15.2 years, *p* = 0.001), had a higher percentage of females (66.0% vs. 46.8%, *p* = 0.013), a higher percentage of the history of prior abdominal operation (61.7% vs. 6.9%, *p* = <0.001), and a higher hemoglobin level (9.3 (8.3–9.8) vs. 8.7 (8.0–9.6) g/dL, *p* = 0.034) as compared to those in the non-IAA group. These two groups had no statistical differences in body mass index, cause of ESRD, and other preoperative laboratory data.

### 3.3. Comparisons of Operative Details and Clinical Outcomes

Table 3 shows the comparisons of operative details and clinical outcomes between two study groups. As shown, the median operative time was found to be 18 min longer in patients who underwent laparoscopic adhesiolysis than in those who did not (93 (80–106) mins vs. 75 (64–87) mins, *p* < 0.001). There were no significant differences in break-in period and other adjunctive procedures between these two groups. Regarding the PD adequacy, the daily dialysate volume was significantly smaller in the adhesiolysis group at both the first (6.0 (6.0–7.2) vs. 6.8 (6–8.5), *p* = 0.002) and second tests (6.2 (6.0–9.0) vs. 8.0 (6.0–10.0), *p* = 0.006). Additionally, a significantly higher renal Kt/V urea was found in the adhesiolysis group at the first (0.78 (0.46–1.06) vs. 0.50 (0.26–0.82), *p* = 0.003) and second tests (0.54 (0.18–0.78) vs. 0.35 (0.15–0.63), *p* = 0.037). However, the dialysate Kt/V urea, total Kt/V urea, dialysate WCCr, renal WCCr, and total WCCr were not significantly different between the two study groups.

**Figure 1:**
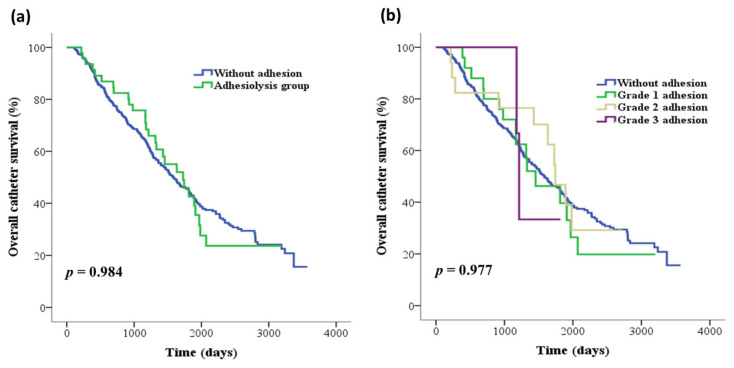
Kaplan–Meier curves of the overall catheter survival for (**a**) patients in the adhesiolysis group versus the non-IAA group and (**b**) patients with different grades of adhesion severity. IAAs, Intra-abdominal adhesions.

The patients in the adhesiolysis group did not experience a higher incidence of catheter obstruction (4.3% vs. 6.1%, *p* = 1.000) than those in the non-IAA group. During follow-up, there was no significant difference in the overall catheter survival between the two study groups (log-rank test, *p* = 0.984) (Figure 1a) or among patients with different grades of adhesion severity (log-rank test, *p* = 0.977) (Figure 1b). Furthermore, no adhesiolysis-related complications, including intra-abdominal organ injury, bleeding, or dialysate leakage, were found during the study period.

## 4. Discussion

The major finding of this study is that laparoscopic adhesiolysis in patients with IAAs confers clinical benefits in achieving PD-related outcomes comparable to those in patients without IAAs. This finding was based on evidence from the outcomes, including the incidence rate of mechanical obstruction, PD adequacy, and overall catheter survival.

It is known that IAAs represent a major problem in patients requiring PD by producing compartmentalization of the abdominal cavity [7,8,10]. This may lead to several consequences, including impeded catheter insertion, tubing mal-positioning, blocked drainage holes resulting in flow dysfunction, and reduced dialyzable space [6,7,8,9,10,11]. These issues may increase catheter insertion difficulty, compromise membrane transport, and unfavorably affect catheter survival [8,9,11,14]. Cheng et al. [6] reported that, compared to patients without IAAs, patients with IAAs had decreased PD adequacy, with no difference in the development of technical failures during PD maintenance. Keshvari et al. [14] reported that there were no significant differences between patients with and without IAAs for overall catheter survival and mechanical dysfunction. However, in their study [14], some patients with IAAs were selected to receive laparoscopic adhesiolysis, to insert a catheter into the uninvolved side, or to create a window for positioning the catheter. As such, it is possible that the negative impact of IAAs was offset by their adjunctive procedures. Nevertheless, these studies suggest the need to treat IAAs during PD catheter implantation to reduce catheter dysfunction, as recommended in the guidelines [8,13].

While many investigators reported performing laparoscopic adhesiolysis in selected patients [12,14,17,18,19], only one study explored the clinical benefits of laparoscopic adhesiolysis in patients requiring PD. Crabtree and Burchette [11] reported that survival probability free from catheter obstruction was lower, but the long-term catheter survival was similar between patients requiring adhesiolysis and those not requiring adhesiolysis, with or without prior abdominal surgery. In contrast to their findings, we found no significant difference in the incidence rate of mechanical obstruction between the adhesiolysis group and the IAA group. In this study, we additionally evaluated PD adequacy, measuring at 12 weeks and 6 months after commencing PD, an outcome that is likely to be perturbed by IAAs [6,7,8,9,10,11]. Our findings regarding the favorable outcome of PD adequacy suggest that adhesiolysis effectively eliminates compartmentalization, restoring the lower abdominal cavity and pelvis to an open space and establishing a sufficient dialyzable area [8,9]. In fact, we found that patients with adhesiolysis had a lower daily dialysis volume compared to patients without IAAs, while achieving a comparable total Kt/V urea. Of note, concerns have been raised about the reformation of abdominal adhesions 6 months after laparoscopic adhesiolysis [29,30]. In this study, the PD adequacy of the second test was well maintained at a level similar to that of the first test, suggesting no reformation of IAAs.

In this study, 61.7% of our patients with a history of previous abdominal surgery had IAAs, an incidence close to the reported range (approximately 70–90%) [5,10]. The top two surgery types were hysterectomy or myomectomy as well as nephrectomy with or without ureterectomy. Our finding is consistent with the observations that a significant portion of patients with IAAs had no prior history of abdominal surgery [6,14,15]. This is due to the fact that other conductions, including inflammation, infection, and radiation therapy, may also produce IAAs [3,4,5]. Since IAAs are not readily detectable preoperatively [4], blind PD catheter insertion in patients with existing IAAs may be complicated by visceral injury, hemorrhage, immediate catheter migration, and catheter malposition with flow dysfunction [12,13,14]. With the emergence of the laparoscopic approach in PD catheter placement as a preferred method [8,9,13], our findings support the advantage of using this technique to directly visualize IAAs if they exist and simultaneously perform adhesiolysis to avoid catheter dysfunction [8,9,13]. This adjunctive procedure inevitably caused an increase in the operative time (about 18 min in our study), which is considered reasonable. As with any surgery, laparoscopic adhesiolysis is associated with the risk of certain complications, including injury to the surrounding organs, infection, and bleeding. However, none of our patients in the adhesiolysis group suffered adhesiolysis-related complications.

Adhesiolysis is an adjunctive procedure of the advanced laparoscopic technique [8,9,13,16]. An early study [31] reported a simplified one-port laparoscopic technique of PD catheter placement with intra-abdominal fixation, and an additional port was used for adhesiolysis in certain patients. Regarding the advanced laparoscopic technique, other proactive adjunctive procedures have also been shown to significantly improve catheter outcomes. For example, Hauch et al. [20] showed that more adjunctive procedures were required in patients with previous abdominal surgery, including adhesiolysis and hernia repair. These adjunctive procedures confer clinical benefits in achieving postoperative catheter complications and overall one-year catheter survival rate in patients with previous abdominal surgery that were comparable to those without surgery history. Crabtree and Burchette [21] reported that they performed adjunctive procedures, including prophylactic omentopexy, adhesiolysis, and hernia repair, in selected patients who underwent PD catheter placement. They concluded that their laparoscopy technique produced superior outcomes in their cohort. Crabtree and Fishman [22] performed 200 catheters implanted using advanced laparoscopic methods, including rectus sheath tunneling, selective prophylactic omentopexy, and selective adhesiolysis. They found that mechanical flow obstruction, the major outcome indicator, followed only 1 of 200 (0.5%) implantation procedures in the advanced group and was significantly better than the open dissection (17.5%) and basic laparoscopic (12.5%) groups. They suggested that catheter mechanical dysfunction attributable to the surgical technique can nearly be eliminated through adjunctive procedures, made possible only by using a laparoscopic approach. Krezalek et al. [23] compared the outcomes among peritoneal dialysis catheter insertions using the open technique, basic laparoscopy with selective adhesiolysis, and advanced laparoscopy utilizing rectus sheath tunnel, selective omentopexy, and adhesiolysis. They showed that the advanced laparoscopy group had the highest rate of dysfunction-free and overall catheter survival. Additionally, the rate of switch to hemodialysis was significantly lower in the advanced laparoscopic group. One recent study [27] compared the outcomes between patients receiving PD catheter placement with and without routine laparoscopic examination for occult inguinal hernias. They concluded that routine laparoscopic examination for occult inguinal hernias with a synchronous repair confers clinical benefits in reducing the risk of developing inguinal hernias after starting PD and the need for a metachronous repair. A more recent study [28] conducted a comparative investigation to assess the benefit/risk profile of routinely performing sutureless omentopexy during laparoscopic PD catheter placement. The authors found that the patients in the non-omentopexy group had a higher incidence of omental wrapping, whereas no patient in the omentopexy group experienced this complication. The authors concluded that this adjunctive procedure confers clinical benefits in reducing the risk of catheter dysfunction due to omental wrapping. Collectively, the findings from the present study and the above-mentioned studies provide clinical evidence that the advanced laparoscopic technique is beneficial to patients receiving PD catheter placement.

There were some limitations in the current study. First, this was a retrospective study with a relatively small patient sample size from a single institution. Future prospective investigations with a larger sample size for a longer follow-up duration are warranted. Second, the adhesion grade was classified as relatively subjective and did not reflect the range of the affected peritoneal area. Third, we did not have data from patients who had IAAs but did not receive laparoscopic adhesiolysis for comparison. This obstacle is due to the protocol set by our hospital that adhesiolysis is a standard procedure after laparoscopic confirmation of IAAs, unless there are contraindications.

## 5. Conclusions

Laparoscopic adhesiolysis in patients with IAAs confers clinical benefits in achieving PD-related outcomes comparable to those in patients without IAAs. This is a safe and reasonable approach. Our findings provide new evidence to support the benefits of this laparoscopic approach, especially in patients with a risk of IAAs.

## Figures and Tables

**Table 1:** Types of prior abdominal operations on patients in the adhesiolysis group.

Type of Prior Surgery	Number of Patients (%)
Hysterectomy or myomectomy *	9 (27.2)
Nephrectomy with or without ureterectomy *	6 (18.2)
Caesarean section	4 (12.1)
Appendectomy *	4 (12.1)
Colectomy	2 (6.1)
Peritoneal dialysis catheter insertion *	3 (9.1)
Gastric surgery *	2 (6.1)
Cholecystectomy *	1 (3.0)
Unknown laparotomy Total	2 (6.1) 33 (100)

* The adhesiolysis group had 29 patients and 4 of them had two prior surgeries.

**Table 2:** Comparisons of clinical characteristics and preoperative laboratory data between the two study groups.

	Adhesiolysis Group (*n* = 47)	Non-IAA Group (*n* = 393)	*p* Value
Age (years)	57.7 ± 14.0	50.2 ± 15.2	0.001
Gender			
Male	16 (34.0)	209 (53.2)	0.013
Female	31 (66.0)	184 (46.8)	
Body mass index (kg/m^2^)	24.1 (20.4–26.8)	23.6 (20.4–26.5)	0.603
Cause of ESRD			0.653
Glomerulonephritis	13 (27.7)	135 (34.4)	
Diabetes mellitus	20 (42.6)	129 (32.8)	
Hypertensive	2 (4.3)	33 (8.4)	
Obstructive nephropathy	1 (2.1)	8 (2.0)	
Polycystic renal disease	2 (4.3)	9 (2.3)	
Others	9 (19.1)	79 (20.1)	
History of abdominal surgery	29 (61.7)	27 (6.9)	<0.001
Preoperative laboratory exam			
BUN (mg/dL)	102.6 (83.5–121.4)	100.0 (7.5–126.9)	0.809
Creatinine (mg/dL)	10.0 (8.4–12.0)	10.4 (8.8–13.1)	0.237
eGFR (mL/min/1.73 m^2^)	4.3 (3.8–5.3)	4.5 (3.7–5.8)	0.587
Albumin (g/dL)	3.66 ± 0.62	3.53 ± 0.58	0.172
Hemoglobin (g/dL)	9.3 (8.3–9.8)	8.7 (8.0–9.6)	0.034
Platelet count (1000/μL)	182 (149–232)	193 (150–244)	0.370
ALT (U/L)	13.5 (9.0–24.0)	13.0 (9.0–22.0)	0.998
Sodium (mEq/L)	137 (134–141)	137 (134–140)	0.410
Potassium (mEq/L)	4.2 (3.7–4.8)	4.3 (3.9–4.8)	0.263
Calcium (mg/dL)	8.4 (7.1–8.8)	8.1 (7.4–8.6)	0.260
Phosphorus (mg/dL)	6.0 (5.2–7.1)	6.3 (5.1–7.6)	0.282

IAA, intra-abdominal adhesion; ESRD, end stage renal disease; BUN, blood urea nitrogen; eGFR, estimated glomerular filtration rate; ALT, aspartate transaminase. Data are presented as mean ± standard deviation, n (%) or median (interquartile ranges).

**Table 3:** Comparisons of operative details and clinical outcomes between two study groups.

	Adhesiolysis Group (*n* = 47)	Non-IAA Group (*n* = 393)	*p* Value
Operative time (minute)	93 (80–106)	75 (64–87)	<0.001
Break-in period (day)	10 (6–11)	10 (8–12)	0.426
Other adjunctive procedure			
Internal fixation	47 (100.0)	393 (100.0)	1.000
Omentopexy	10 (21.3)	67 (17.0)	0.471
Inguinal hernia repair	1 (2.1)	18 (4.6)	0.708
Umbilical hernia repair	0 (0.0)	1 (0.3)	1.000
Salpingo-oophorectomy	0 (0.0)	4 (1.0)	1.000
Catheter obstruction	2 (4.3)	24 (6.1)	1.000
1st PD adequacy test			
Daily dialysate volume (L)	6.0 (6.0–7.2)	6.8 (6–8.5)	0.002
Dialysate Kt/V urea	1.41 (1.20–1.61)	1.49 (1.24–1.76)	0.063
Renal Kt/V urea	0.78 (0.46–1.06)	0.50 (0.26–0.82)	0.003
Total Kt/V urea	2.13 (1.89–2.48)	2.06 (1.78–2.39)	0.597
Dialysate WCCr (L/W/1.73 m^2^)	34.4 (27.4–39.7)	36.9 (30.7–44.4)	0.046
Renal WCCr (L/W/1.73 m^2^)	31.6 (20.9–47.7)	30.0 (14.9–44.3)	0.157
Total WCCr (L/W/1.73 m^2^)	64.9 (52.3–78.9)	67.5 (54.6–83.1)	0.685
2nd PD adequacy test *			
Daily dialysate volume (L)	6.2 (6.0–9.0)	8.0 (6.0–10.0)	0.006
Dialysate Kt/V urea	1.64 (1.36–1.81)	1.63 (1.45–1.91)	0.384
Renal Kt/V urea	0.54 (0.18–0.78)	0.35 (0.15–0.63)	0.037
Total Kt/V urea	2.17 (1.88–2.42)	2.05 (1.80–2.37)	0.174
Dialysate WCCr (L/W/1.73 m^2^)	38.7 (31.1–44.2)	41.6 (33.5–48.0)	0.053
Renal WCCr (L/W/1.73 m^2^)	24.4 (10.8–35.9)	19.0 (9.1–34.4)	0.214
Total WCCr (L/W/1.73 m^2^)	56.6 (50.1–72.3)	62.6 (50.6–75.2)	0.568

Kt/V, [(dialyzer clearance of urea) × (dialysis time)]/(volume of distribution of urea); IAA, intra-abdominal adhesion; PD, peritoneal dialysis; WCCr, weekly creatinine clearance. Data are presented as n (%) or median (interquartile ranges). Two PET and PD adequacy tests were performed at 12 weeks and 6 months after commencing PD. * Three patients in the adhesiolysis group and 33 patients in the non-IAA group did not complete the 2nd PD adequacy test.

## Data Availability

All data involved in this study will be made available by the corresponding author upon request.
